# Dermatofibrosarcoma protuberans: clinicopathologic presentation in Nigerians

**DOI:** 10.11604/pamj.2018.31.25.13665

**Published:** 2018-09-12

**Authors:** Olajumoke Ajibola Effiom, Akanbi Clement Olurotimi Olojede, Olakanmi Ralph Akinde, Adetokunbo Babjide Olawuyi, Abiodun Taofeek Amoo, Godwin Toyin Arotiba

**Affiliations:** 1 Department of Oral and Maxillofacial Pathology/Biology, College of Medicine, University of Lagos, Nigeria; 2 Department of Oral and Maxillofacial Surgery, College of Medicine University of Lagos, Nigeria; 3 Department of Anatomic and Molecular Pathology, College of Medicine, University of Lagos, Nigeria; 4 Department of Oral and Maxillofacial Pathology/Biology, Lagos University Teaching Hospital, Nigeria; 5 Department of Oral and Maxillofacial Surgery, Lagos University Teaching Hospital, Nigeria

**Keywords:** Dermatofibroma sarcoma protuberance, facial, Nigerians

## Abstract

**Introduction:**

Dermatofibrosarcoma protuberance (DFSP) is in general a rare low grade malignant sarcoma and possesses a tendency for local recurrence. It has a site predilection for the trunk. Occurrence in the facial area is extremely rare. Ample knowledge of its clinical, histological and biologic characteristics is vital for accurate and prompt recognition.

**Methods:**

Over 13 years, clinicohistologic information of cases was retrieved. Histological and immunohistochemical re-evaluation were performed to re-confirm diagnosis. Data collected and analyzed with SPSS Statistics version 20 were presented as frequency tables, charts and proportions as appropriate.

**Results:**

Of 191 soft tissue sarcomas, a total of 28 cases were diagnosed as DFSP (14.7%). Facial types occurred in 3 cases (1.6%). Tumour had age and site predilections for the 4^th^ decade and trunk respectively. There was an equal gender distribution among cases. Most common clinical presentation was in form of painless protruding nodular mass. General histologic presentation revealed cellular lesions composed of spindle to oval neoplastic cells arranged in a storiform pattern. Mitotic figures were rare. All cases showed positive expressions to CD34.

**Conclusion:**

Facial DFSP is rare among Nigerians. Its clinical appearance may mimic other common benign lesions of the head and neck region often resulting in misdiagnoses. A comprehensive knowledge of its clinical and histologic presentations and biologic behavior, combined with its identification with the aid of advanced histologic and radiographic techniques results in prompt confirmatory diagnosis. Appropriate treatment should include adequate surgical excision techniques combined with adjuvant radiotherapy or chemotherapy.

## Introduction

Dermatofibrosarcoma protuberans [DFSP] is a rare superficial soft tissue sarcoma. It constitutes less than 0.1% of all malignant neoplasms and about 1.0% of all soft tissue sarcomas worldwide. Though uncommon, it is the most common sarcoma that originates from the skin [1]. It has a site predilection for the trunk and extremities, and facial involvement, though quite seldom, have been reported [1]. DFSP is regarded as a sarcoma which in general possesses a low grade malignant aggressive biologic behavior. It has a low metastatic potential and the tendency for local recurrence post treatment [1]. Although its precise etiology remains unknown, it has been linked to chromosomal translocation of t (17;22) (q22; q13) [1]. Clinically, DFSP begins as an asymptomatic lesion that increases slowly in size. Over time it enlarges and may structurally contain protruding nodules. Its earliest description was by Sherwell and Taylor in 1890 [1]. Darrier et al [2] subsequently labelled it “recurrent progressive dermatofibroma” but the name dermatofibrosarcoma protuberans was coined by Hoffman in 1925 [3]. Reports on the clinic pathological presentations of DFSP among Nigerians are rare with even rarer reports on facial DFSP cases. This may result in non-recognition of DFSP or its delayed diagnosis with subsequent misdiagnosis or delayed treatment respectively. We therefore aim to elucidate its clinic pathological presentation in order to aid early diagnosis. Data from the present study would in addition update existing data in the scientific literature.

## Methods

From the oral biopsy record files of the Oral and Maxillofacial Pathology /Biology and Anatomic and Molecular Pathology Departments of the Lagos University Teaching Hospital, all cases that had been previously diagnosed as DFSP over a 13-year period, were identified. Hematoxylin and eosin (H and E) glass slides and immunohistochemical slides (using CD34, S100 and Vimentin markers) of the identified cases were retrieved and reviewed to re-confirm diagnosis of DFSP. Clinical data regarding age, gender, location and treatment were obtained and compiled. Estimated size of each lesion/month was computed using the method by Effiom and Odukoya [4]. This was analyzed using the estimated volume of each tumor at time of presentation to compute each estimated tumor volume/month. The estimated volume of tumor was computed using the equation 4/3 x 22/7x radius^3^ (radius being ½ of diameter that was recorded for each tumor) based on the assumption of the tumor being spherical. The estimated tumor size at presentation was further categorized into 3 main sizes namely: large- for tumor volume sizes at presentation greater than 500cm^3^, medium- for tumor volume sizes that range between 100 and 500 cm^3^ and small-for tumor volume sizes less than 100cm^3^. Likewise, the estimated tumor volume/month was categorized into possibly fast growth- for values greater than 1.0cm^3^, possibly medium growth- for values between 0.5cm^3^ to 1.0cm^3^ and possibly slow growth- for values less than 0.5cm^3^. Data was analyzed using the statistical package for social sciences software package for windows version 20 and these were presented as ranges, percentages, median, mean, standard deviations and tables as appropriate.

## Results

**Figure 1:**
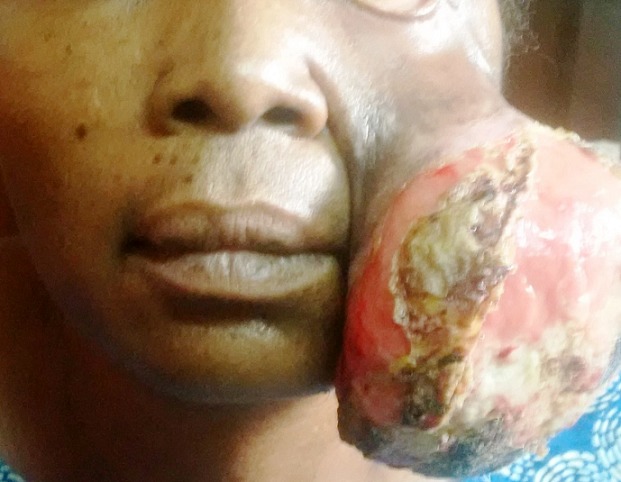
A case of an extensive facial DFSP in a Nigerian 48 year old female. Note the extensive facial asymmetry on the left side of the face, ulcerative bleeding surface of the protruding mass and ectropion of the left lower eye lid

**Figure 2:**
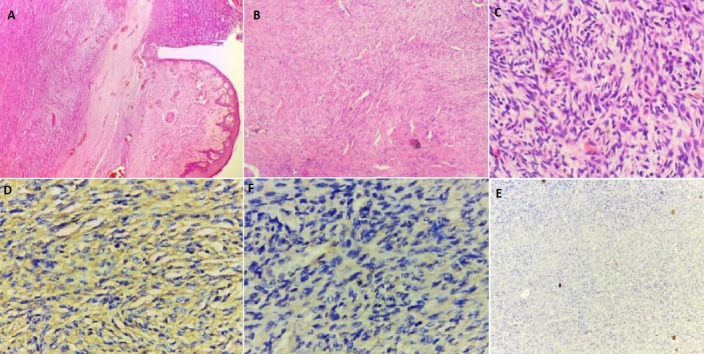
( A B, C, D) photomicrograph of DFSP: (A) higher magnification showing spindle cells in connective tissue stroma (H&E X40); (B) CD 34 positive cytoplasmic staining of tumor cells in DFSP. Positive areas show as brown colorations; (C) Diffuse staining with vimnetin. Brown coloration is indicative of positive areas; (D) Negative staining with S-100

A total of 191 soft tissue sarcomas were reported over a 13 -year period. DFSP occurred in 28 subjects (14.7%) while facial DFSP occurred in only 3 cases (1.6% of soft tissue sarcomas and 11.0% of all DFSP). Age ranged between 1-80 years with an equal sex predilection (Table 1). DFSP had an age predilection for subjects in the 4^th^ decade of life in the present series. The mean age of subjects at presentation was 36.43 ± 16.4. Mean duration was 34.91 months ± 21.16. DFSP had a site predilection for the trunk (Table 1). DFSP with facial locations (facial DFSP) occurred more in females (Figure 1) and among subjects within 24 and 48 years of age. Majority of the DFSP at hospital appearance, presented as painless multinodular protruding masses, with few being ulcerated and hemorrhagic. We however observed that 1 case presented as a painless keloid-like mass (Table 1). Tumor volume at presentation which ranged from 0.5cm^3^ to 4176.2cm^3^ (mean =1238.42cm^3^, ±2554.67) was computed in 19 subjects. estimated monthly tumor volume ranged from 0.02cm^3^/month to 285.4 cm^3^/month (32.56cm^3^ ±15.26) (Table 2). Histologic H&E tissue examination (Figure 2 A) basically revealed cellular lesions (ranging from moderate to highly cellular lesions) composed of spindle to oval neoplastic cells arranged in a storiform pattern. The neoplastic cells infiltrated into subcutaneous tissue in varying degrees but in all mitotic figures were scarce. We therefore made diagnoses of mesenchymal soft tissue tumors, consistent with conventional DFSP. Immunohistochemical re-evaluation (Figure 2 B, C, D) showed positive expressions to CD 34 in all cases (Figure 2 B). Specifically, immunohistochemical re-evaluation of the 3 cases of facial DFSP showed strong positive expressions to CD34, weak but diffuse positive expressions to vimentin in 2 facial cases (Figure 2 C) and negative reactions to S-100 (Figure 2 D). Definitive diagnoses of DFSP were made for all 28 cases.

**Table 1:** Clinical pattern of presentation of 28 cases of DFSP

Age (years)	Gender	Site	Patient description of lesion at first appearance	Clinical appearance of lesion at hospital presentation
80	Male	Left foot-lower extremity	Painless single swelling	Ulcerated pedunculated mass with multiple nodular surface
44	Female	Right thigh-lower extremity	Painless single firm swelling	Painless protruding mass
35	Male	Right shoulder-trunk	N/A	Painless protruding mass with multi nodular surface
70	Female	Left foot-lower extremity	Painless single swelling	Firm Painless protruding mass with Nodular surface
25	Female	Right gluteal-buttock	N/A	Painless Multinodular protruding mass
26	Male	Back-trunk	Boil	Ulcerated painful protruding nodular mass
39	Female	Right thigh-lower extremity	Painless Nodule	Painless Multinodular protruding mass
25	M ale	Back-trunk	Boil	Painful Ulcerated multinodular protruding mass
18	Male	Left leg-lower extremity	Boil	Painless Ulcerated multinodular protruding mass
38	Male	Left shoulder- trunk	Painless Nodule	Painless hemorrhagic protruding mass with multinodular surface
40	Male	Anterior Abdominal wall-trunk	Painless firm Nodule	protruding Multinodular mass
30	Male	Left thigh-lower extremity	N/A	Painful Ulcerated protruding multinodular mass
41	Female	Right foot-lower extremity	Boil	Painless Multinodular hemorrhagic protruding mass
38	Male	Left shoulder-trunk	Painless scar	Painless keloid- like swelling
50	Female	Anterior Abdominal wall -trunk	Painless firm Nodule	Painless Ulcerated multinodular protruding mass
16	Female	Left leg-lower extremity	Painless Single Nodule	Painless firm protruding mass with Nodular surface
43	Male	Upper arm-upper extremity	Painless Nodule	Painless firm protruding mass with Nodular surface
8	Female	Back-trunk	Painless Nodule	Painless protruding mass
40	Male	Preauricular (middle 3rd of face)	N/A	Painless firm protruding mass with Nodular surface
40	Male	Anterior Chest wall-trunk	Painless firm swelling	Painless protruding mass
29	Female	Right leg-lower extremity	Painless swelling	Painless firm protruding mass with Nodular surface swellings
1	Female	Anterior Abdominal wall-trunk	Painless soft swelling	protruding Nodular swelling
40	Male	Back-trunk	N/A	Painless Ulcerated protruding mass with nodular swellings
24	Female	Angle of mouth (middle 3rd of face)	N/A	Painless hemorrhagic multinodular protruding mass
38	Female	Back-trunk	Firm painless Nodule	Painless protruding Nodular swelling
52	Female	Leg-lower extremity	Painless Nodule	Painless Ulcerated protruding nodular mass
48	Female	Left side of face middle and lower 3rd of face	Boil	Painless ulcerated hemorrhagic protruding mass
42	Male	Left leg-lower extremity	N/A	Painless Multinodular protruding mass

NS = Not stated in the biopsy files, N/A= not available. All cases were treated by wide surgical excision procedure

**Table 2:** Size distribution of DFSF

Age (years)	Gender	Site	Estimated largest diameter of lesion(cm)	Radius x3 (cm)	Estimated duration of lesion in months	Estimated volume of lesion( cm 3) 4/3x22/7xr3	estimated volume of lesion /month ( cm3/month ) mean =32.56cm3±66.6	Estimated largest diameter of lesion(cm)
80	Male	Left foot-lower extremity	2.0	1.0	24	4.2	0.2	2.0
44	Female	Right thigh-lower extremity	3.0	3.4	24	14.1	0.6	3.0
35	Male	Right shoulder-trunk	1.5	0.4	36	1.8	0.1	1.5
70	Female	Left foot-lower extremity	NS	NA	NS	NA	NA	NS
25	Female	Right gluteal-buttock	9.0	91.1	30	380.5	12.7	9.0
26	Male	Back-trunk	20.0	1000.0	48	4176.2	87.0	20.0
39	Female	Right thigh-lower extremity	NS	NA	NA	NA	NA	NS
25	M ale	Back-trunk	16.0	512.0	48	2138.2	45.0	16.0
18	Male	Left leg-lower extremity	15.0	422.0	NA	NA	NA	15.0
38	Male	Left shoulder- trunk	27.0	2460.0	36	10275	285.4	27.0
40	Male	Anterior Abdominal wall-trunk	5.0	15.6	24	65.25	2.7	5.0
30	Male	Left thigh-lower extremity	NS	NA	30	NA	NA	NS
41	Female	Right foot-lower extremity	NS	NA	36	NA	NA	NS
38	Male	Left shoulder-trunk	NS	NA	NA	NA	NA	NS
50	Female	Anterior Abdominal wall -trunk	20.0	1000.0	120	4176.2	34.8	20.0
16	Female	Left leg-lower extremity	7.0	42.9	24	179.1	7.5	7.0
43	Male	Upper arm-upper extremity	11.0	166.4	24	694.8	29.1	11.0
8	Female	Back-trunk	1.0	0.1	24	0.5	0.02	1.0
40	Male	Preauricular (middle 3rd of face)	2.5	2.0	36	8.2	0.23	2.5
40	Male	Anterior Chest wall-trunk	5.0	15.6	24	65.3	2.7	5.0
29	Female	Right leg-lower extremity	8.0	64.0	36	267.3	7.4	8.0
1	Female	Anterior Abdominal wall-trunk	1.0	0.1	24	0.5	0.02	1.0
40	Male	Back-trunk	NS	NA	NA	NA	NA	NS
24	Female	Angle of mouth (middle 3rd of face)	1.5	0.4	36	1.8	0.1	1.5
38	Female	Back-trunk	NS	NA	24	NA	NA	NS
52	Female	Leg-lower extremity	7.0	43.0	48	179.1	3.7	7.0
48	Female	Left side of face middle and lower 3rd of face	12.0	216.0	12	902.1	75.2	12.0
42	Male	Left leg-lower extremity	NS	NA	NA	NA	NA	NS

NS = Not stated in the biopsy files, N/A= not available. All cases were treated by wide surgical excision procedure

## Discussion

DFSP is a rare, low grade, slow growing fibrohistiocytic malignancy that arises from the dermis, leaves a Grenz zone but extends into subcutaneous tissue [5-7]. It is the most common cutaneous sarcoma and constitutes less than 0.1% of all malignant neoplasms and about 1% of all soft tissue sarcomas worldwide [8]. In Nigeria, it has been reported to account for approximately 7.0% of soft tissue sarcomas over a 22-year period of study [9]. DFSP represented 14.7% of reported cases of soft tissue sarcomas in our series, which may indicate low prevalence among Nigerians. Perusal of the scientific literature shows variation in gender pattern of the tumor [6, 9]. It majorly occurs in adults within a wide age range, having a predilection for the 2^nd^ to 5^th^ decades of life [6]. Similarly, we also report a wide age range with a mean age of 36 ± 16.4.years Though it most commonly occurs in the trunk and the upper and lower extremities [2, 3, 5, 6]. DFSP have been reported to seldom occur in the head and neck region and specifically even more rarely in the facial region [6, 9]. Approximately 10-15% of cases have been reported to account for head and neck DFSP. There have been reports of 3 and 2 facial locations (facial DFSP) from 25 and 86 cases of DFSP respectively in the scientific literature [6]. Similarly, we report 3 facial DFSP from 28 cases of DFSP. Diagnostic challenges amongst pathologist and surgeons do occur with cases of DFSP. Early clinical appearances of DFSP may mimic the appearances of other common benign lesions [10-12] which may result in misdiagnoses. While DFSP typically presents as an asymptomatic slow growing lesion, there is variance in its clinical presentation. It may initially present as a hard or firm indurated plaque, scar or protruding mass [1, 7] therefore mimicking common benign lesions such as Keloids, cysts boils etc. With time, the lesion may develop multiple nodules, which justifies the addition of the word “protuberans” to its original name of dermatofibrosarcoma [**1**]. We observed similar pattern of presentation. At first appearance, some cases were described as “boils” and “scar” by subjects.

Clinical size of DFSP has frequently been reported to range from 2-5cm in diameter although large tumors have been stated. We also report several huge sized DFSP with diameters >5cm (range of tumor diameter of cases = 1.0-27.0cm) and estimated tumor volumes (though hypothetical) as small as 0.5cm^3^ to sizes as enormous as 10275cm^3^. Most of the enormous sized DFSP in particular, initially appeared as simple “boils” (a misdiagnosis) which were probably not considered as important lesions for prompt treatment. Besides, such lesions could have been inappropriately managed over time due to misdiagnosis. Delay in appropriate treatment in Nigerians could be a factor responsible for huge tumor sizes. From the computed estimated tumor volume/month (though hypothetical) majority of tumors in our series appear to have a fast rather than a slow growing biologic nature reported in the literature. Future studies that determine the biologic nature of DFSP may be conducted. Usually DFSP is fixed to overlying skin but not fixed to the underlying deeper structures. Invasion into underlying deeper structures such as the muscles, bone fascia occur with more aggressive histologic variants or longstanding recurrent tumors [8, 10]. All lesions in the present series were not fixed to deeper structures which is a feature that implies a low grade invasive growth. Few cases were however extensively ulcerated and hemorrhagic. Some were in addition painful. These clinical presentations could be attributed to infections from inappropriate management such as application of herbal concoctions or some form of self-medication overtime. It may however on the other hand, be indicative of the sarcomatous nature of the tumor. DFSP is regarded as having a “low grade” biologic aggressive nature. Though viewed as locally aggressive, it has been reported to possess some metastatic potential. It is therefore required that clinicians use advanced radiographic imaging techniques such as Magnetic resonance imaging (MRI) and CT Scan to assess tumor extent. Lung and bone Metastasis have been reported in about 3% of cases [7, 10]. Though unspecific, tomographic imaging generally show the presence of intermediate to high enhancement on contrast of well-defined homogenous soft tissue mass of DFSP.

Conventional histologic type of DFSP has been described as a circumscribed lesion that occupies the whole dermis. It is composed of spindle cells usually arranged in a storiform pattern within a moderately collagenized stroma [8]. The tumor has been described as being highly cellular with few mitotic figures [6]. Nodular lesions have been observed to have more prominent features of cellular atypia and mitotic figures than plaque lesions. There have been reports of fibrosarcomatous transformation of DFSP associated with a more aggressive tumor [6, 8]. It is also important to note the existence of various histologic sub types of DFSP. The lack of recognition of these subtypes may result in histologic misdiagnosis and inappropriate management. For example, DFSP with fibrosarcomatous areas subtype may be misdiagnosed as fibrosarcoma. Proper characterization and recognition of the various histologic types is therefore imperative to avoid misdiagnosis. Other histologic types of DFSP include: pigmented, myxoid, granular cell, sclerotic, atrophic DFSP, giant cell fibroblastoma, and of DFSP [1] . A definitive diagnosis of DFSP should be made with the use of immunohistochemical analysis. Even though CD34 has been reported to be positively expressed with some cases of angiosarcoma and myofibrosarcoma [13], 90% of DFSP cases show positive reaction to CD34. [1] In addition, DFSP show positive reaction to PDGFR-B and in some cases to vimentin [6, 7]. DFSP has been observed to show negative reaction to EMA, smooth muscle actin, CD31, cytokeratin5/6, desmin and in some cases, alpha XIII a [1, 7]. All cases of the Facial DFSP showed positivity for vimentin and CD34 which confirmed a diagnosis of DFSP. Treatment of DFSP especially the plague/scar like DFSP, is by surgery with Mohs micrographic surgery (MMS) providing better treatment outcomes than Wide surgical resection (WSR) due to its lower recurrence rates as well less disfigurement and functional impairment [7, 8]. Huge DFSP tumors can also be treated using wide surgical resection with 0.5 to 1.0cm margin especially in hospitals where the expertise for MMS is limited or non - existent. Reports from previous studies show the use of imatinib mesylate as adjuvant targeted molecular therapy for un-resect able, metastatic or recurrent cases of DFSP [7, 9]. Imatinib mesylate is a potent selective tyrosine kinase inhibitor that inhibits platelet derived growth factor tyrosine kinase DFSP with t (17, 22) (q22; q13) chromosomal translocations has been observed to respond to imatinib mesylate [1, 7]. Long term follow-up is essential because of local recurrences which commonly occur in the first year after surgery though this may also occur after five years post-surgery. There were no available records on recurrence for cases in our series.

## Conclusion

Elucidation of the clinical, histologic and biologic presentation of DFSP is important as this would improve clinician's knowledge of the tumor and allow early recognition. This will result in prompt delivery of appropriate treatment and reduce patient morbidity and mortality.

### What is known about this topic

- DFSP is a relatively rare cutaneous locally aggressive sarcoma;
- Tumor has with some malignant potential;
- Reports on cases especially DFSP on the facial region are rare.

### What this study adds

- Study elucidates the clinicopathologic presentations of DFSP in Nigerians to allow its prompt recognition;
- Study highlights appropriate treatment modalities for effective management of DFSP;
- Data from this study updates existing data on DFSP in the scientific literature.

## Competing interests

The authors declare no competing interest.
